# Identification and Isolation of Cardiac Fibroblasts From the Adult Mouse Heart Using Two-Color Flow Cytometry

**DOI:** 10.3389/fcvm.2019.00105

**Published:** 2019-08-01

**Authors:** Mara Stellato, Marcin Czepiel, Oliver Distler, Przemysław Błyszczuk, Gabriela Kania

**Affiliations:** ^1^Department of Rheumatology, Center of Experimental Rheumatology, University Hospital Zurich, Zurich, Switzerland; ^2^Department of Clinical Immunology, Jagiellonian University Medical College, Cracow, Poland

**Keywords:** cardiac fibroblast, collagen I, gp38/podoplanin, FACS sorting, flow cytometry

## Abstract

**Background:** Cardiac fibroblasts represent a main stromal cell type in the healthy myocardium. Activation of cardiac fibroblasts has been implicated in the pathogenesis of many heart diseases. Profibrotic stimuli activate fibroblasts, which proliferate and differentiate into pathogenic myofibroblasts causing a fibrotic phenotype in the heart. Cardiac fibroblasts are characterized by production of type I collagen, but non-transgenic methods allowing their identification and isolation require further improvements. Herein, we present a new and simple flow cytometry-based method to identify and isolate cardiac fibroblasts from the murine heart.

**Methods and Results:** Wild-type and reporter mice expressing enhanced green fluorescent protein (EGFP) under the murine alpha1(I) collagen promoter (Col1a1-EGFP) were used in this study. Hearts were harvested and dissociated into single cell suspensions using enzymatic digestion. Cardiac cells were stained with the erythrocyte marker Ter119, the pan-leukocyte marker CD45, the endothelial cell marker CD31 and gp38 (known also as podoplanin). Fibroblasts were defined in a two-color flow cytometry analysis as a lineage-negative (Lin: Ter119^−^CD45^−^CD31^−^) and gp38-positive (gp38^+^) population. Analysis of hearts isolated from Col1a1-EGFP reporter mice showed that cardiac Lin^−^gp38^+^ cells corresponded to type I collagen-producing cells. Lin^−^gp38^+^ cells were partially positive for the mesenchymal markers CD44, CD140a, Sca-1 and CD90.2. Sorted Lin^−^gp38^+^ cells were successfully expanded *in vitro* for up to four passages. Lin^−^gp38^+^ cells activated by Transforming Growth Factor Beta 1 (TGF-β1) upregulated myofibroblast-specific genes and proteins, developed stress fibers positive for alpha smooth muscle actin (αSMA) and showed increased contractility in the collagen gel contraction assay.

**Conclusions:** Two-color flow cytometry analysis using the selected cell surface antigens allows for the identification of collagen-producing fibroblasts in unaffected mouse hearts without using specific reporter constructs. This strategy opens new perspectives to study the physiology and pathophysiology of cardiac fibroblasts in mouse models.

## Introduction

Cardiac fibroblasts represent the main stromal cell type in the adult heart, which control extracellular matrix (ECM) turnover and maintain tissue structure (1–3). Fibroblasts are commonly defined as type I collagen-producing cells. For a long time, fibroblasts have been thought to be a homogenous cell population of mesenchymal origin with similar functions in different organs. However, several recent studies have demonstrated that fibroblasts present an extensive phenotypic heterogeneity (4–6). Disbalance in production and degradation of ECM components is associated with tissue remodeling. Activation of cardiac fibroblasts often leads to excessive accumulation of ECM proteins (mainly type I collagen) and proliferation of stromal cells in the myocardium. Fibrosis in the heart causes not only impaired mechanical contraction, but also can disturb conduction of electrical impulses. Progressive fibrosis is considered as one of the major causes of heart failures. This process is regulated by both, intrinsic mechanisms, such as transcriptional regulatory networks (7) and epigenetic processes, and by extrinsic factors, such as cell-to-cell signaling, soluble signaling mediators or ECM components. Transforming growth factor beta (TGF-β) represents a key profibrotic cytokine that activates and differentiates fibroblasts into pathological myofibroblasts. Myofibroblasts are characterized by excessive ECM production and enhanced contractility potentials and their presence is a key feature of cardiac fibrosis (8). Filaments positive for alpha smooth muscle actin (αSMA) is a commonly used marker of myofibroblasts.

A routine method to obtain cardiac fibroblasts is based on their selective adhesion and high expansion potential in cell cultures. Typically, outgrowing cells isolated from cardiac muscle are considered as fibroblasts. Indeed, while using optimized protocols, many cells obtained with this method show features of fibroblasts (2). This method is, however, not specific, and does not allow for analysis of freshly isolated cells.

Flow cytometry and fluorescence-activated cell sorting (FACS) represent widely used technologies to identify and isolate cells from a single cell suspension based on fluorescence. Antibodies conjugated with different fluorescent dyes allow detecting multiple markers simultaneously on a single cell. Analysis of living cells by flow cytometry and FACS requires, however, the use of antibodies recognizing cell membrane markers. Many cell types express specific cell membrane antigens, as for example haematopoietic cells (CD45) or endothelial cells (CD31). Cardiac fibroblasts were characterized by expression of certain cell surface antigens (9), but so far, the unique, fibroblast-defining cell surface marker is not available.

A subset of stromal cells recently identified in secondary lymphoid organs (SLOs) is defined by gp38 (called also podoplanin) positivity (4). At first, gp38 expression was found to be essential during development (10). In the developing heart, gp38 plays an important role in development of sinus venous myocardium (11). In the adult heart, gp38 is expressed in cardiac lymphatic vessels and in interstitial cells (12). Conversely, recent studies demonstrated that gp38 expression is limited to lymphatic endothelial cells and fibroblastic reticular cells, suggesting that gp38^+^ cells may play an important role in different organs during homeostasis and disease processes such as inflammation or fibrosis (5, 13). Flow cytometry study in SLOs demonstrated that fibroblastic cells are negative for hematopoietic and endothelial lineage markers CD45 and CD31 defining a population of lymphatic fibroblasts (14). Characteristic of cardiac gp38^+^ cells with flow cytometry has not been clarified yet. We hypothesized that lineage-negative and gp38^+^ cells in the heart might specifically define a population of cardiac fibroblasts.

## Methods

### Preparation of Single Cell Suspension From Mouse Heart

Wild-type C57BL/6 mice were purchased from Charles Rivers. Col1a1-EGFP (pCol9GFP-HS4,5) reporter mice (15) were kindly provided by Prof. D.A. Brenner, San Diego, USA. All mice were housed in pathogen-free conditions. Animal experiments were performed in accordance with the Swiss federal law and with the Guide for the Care and Use of Laboratory Animals published by the US National Institutes of Health (NIH Publication, 8th Edition, 2011). The Cantonal Veterinary Office in Zurich had approved animal experiments.

Hearts of 6–8-week-old wild-type and Col1a1-EGFP reporter mice were perfused with ice-cold phosphate buffered saline (PBS) through the left ventricle, were harvested and kept in PBS on ice. Hearts were cut into small pieces, resuspended in 0.025 mg/mL of Liberase TM solution (Roche) containing collagenase I and II and DNAse (40 ug/mL, Stemcell Technologies) in pure Dulbecco's modified Eagle's medium (DMEM, Sigma) and incubated for 45 min at 37°C. Enzymatic digestion was supported by mechanical tissue fragmentation using magnetic beads (VWR) and magnetic stirrer. Digested tissue was filtered through a 70 μm cell strainer followed by low speed centrifugation (50 g, 2 min) to remove cardiomyocytes, cell clumps and the remaining undigested tissue. Next, the supernatants were passed through a 40 μm cell strainer, centrifuged at 450 g for 4 min and washed with PBS. Single cell suspension for flow cytometry staining was obtained by resuspending cell pellet in FACS buffer: (PBS supplemented with 1% fetal bovine serum (FBS, ThermoScientific/Life Technology) and 1 mM EDTA (ThermoScientific/Invitrogen).

### Flow Cytometry and Cell Sorting

Cells in single cell suspensions were blocked with anti-mouse CD16/CD32 (1:200; Clone 93, Thermo Fisher Scientific) for 10 min at 4°C, followed by incubation on ice for 30 min with the appropriate combination of fluorochrome-conjugated antibodies diluted in FACS buffer: TER119-PE, (1:600, clone TER-11, eBioscience), CD45-PE (1:300, clone 30-F11, eBioscience), CD31-PE (1:300, clone 390, Thermo Fisher Scientific), gp38-APC (1:100, clone 8.1.1, eBioscience), CD90.2-eFluor 450 (1:300, clone 53-2.1, eBioscience), Sca-1- PerCP-Cy5.5 (1:300, clone D7, eBioscience), CD44-PE-Cy7 (1:300, clone IM7, eBioscience), CD140a-PE-Cy7 (1:300, clone APA5, eBioscience). 4′,6-diamidino-2-phenylindole (DAPI 1 μg/mL, Roche) was used to distinguish live/dead cells. Cells were analyzed with the BD LSR Fortessa flow cytometer (BD Biosciences) and sorted with the FACSAria III 4L (BD Biosciences). FlowJo (version 10.08) was used for data analyses.

### Cell Culture

Sorted Lin^−^gp38^+^ cells were cultured in DMEM (Gibco) supplemented with 10% FBS, 50 U/mL penicillin, 50 μg/mL streptomycin (both Gibco) and 50 mM β-mercaptoethanol (Thermo Fisher Scientific). Cells were passaged up to 4 passages. Cells from passages 3 and 4 were used in differentiation experiments. Cell differentiation was induced with 10 ng/mL mouse recombinant TGF-β1 (Peprotech). All cells were cultured in humified incubators under standard culture conditions at 37°C and 5% CO_2_.

### RNA Extraction and qPCR

Cells were washed with PBS, mixed with RNA lysis buffer (Zymo) and frozen at −80°C. Zymo Quick-RNA MicroPrep isolation kit was used to extract total RNA. 100% ethanol was added to the lysates and samples were than processed on the MicroPrep columns. DNA contamination was removed by DNAse I digestion. RNA was washed and eluted in 10 μl of water. RNA concentration and purity were assessed with Nanodrop 1000 (Thermo Fisher Scientific). Reverse transcription of 125 ng total RNA was performed using the Transcriptor First Strand cDNA Synthesis kit (Roche) and random hexamers, according to the manufacturer's protocol. qPCRs were performed with 2x SYBR Green master mix (Promega) and oligonucleotides for the genes of interest using an Agilent Technologies Stratagene Mx3005P qPCR system. *Acta2* oligonucleotides: forward (5′->3′): CGCTGTCAGGAACCCTGAGA, reverse (5′->3′): CGAAGCCGGCCTTACAGA; *Gapdh* (housekeeping gene) oligonucleotides: forward (5′->3′): CTGCACCACCAACTGCTTAGC, reverse (5′->3′): GGCATGGACTGTGGTCATGAG. Relative *Acta2* expression was normalized to *Gapdh* levels.

### Western Blotting

Cellular proteins were extracted with RIPA buffer (Sigma-Aldrich) supplemented with protease inhibitor cocktail (Complete ULTRA Tablets, Roche) and phosphatase inhibitors (PhosphoStop, Roche) from cultivated cells. Protein concentration was quantified by colorimetric bicinchoninic acid assay according to the manufacturer's protocol (Thermo Fisher Scientific). SDS-PAGE electrophoresis and wet-transfer method were used to separate and transfer proteins on nitrocellulose membranes followed by 45 min incubation in blocking solution: [tris buffered saline, Tween-20 (TBST, Thermo Fisher Scientific) containing 5% skim milk powder (Becton Dickinson AG)]. Membranes were incubated overnight with the following primary antibodies: anti-αSMA (1:1000, clone 1A4, Sigma-Aldrich) or GAPDH (1:10000, clone 14C10, Cell Signaling). Horseradish peroxidase (HRP)-conjugated secondary antibodies were used for detection with ECL substrate (SuperSignal West Pico Plus, Thermo Fisher Scientific) and development on the Fusion Fx (Vilber). Densitometric analyses were performed with ImageJ 1.47t. Fold changes were computed after normalization to GAPDH.

#### ELISA

Lin^−^gp38^+^ cells were cultured for up to 4 passages and seeded with or without TGF-β1 (10 ng/mL) in 1% FBS medium for 7 days. Supernatants were collected and diluted 1:100 for assessing the amount of pro-collagen I alpha 1 using ELISA (Abcam) according to the manufacturer's instructions. The amount of pro-collagen I alpha 1 in the samples was calculated as interpolation of the standard curve.

### Immunofluorescent Staining

#### Cells

Cells were seeded in 8-chamber slides (Lab-Tec) at the density of 2,500 cells/well, fixed in ice-cold methanol:acetone 7:3 (both Sigma-Aldrich) for 10 min at −20°C, washed with PBS and blocked with 10% FBS in PBS for 20 min at room temperature. Subsequently, incubation with the primary mouse anti-αSMA antibody (clone: clone 1A4, 1:100, Sigma-Aldrich) was performed for 1 h at room temperature followed by staining with the secondary Alexa 488 goat anti-mouse antibody (1:400, Thermo Fischer Scientific) and 50 μg/ml of fluorescent labeled phalloidin (Sigma-Aldrich) to visualize stress fibers for 45 min at room temperature. Nuclei were counterstained with DAPI solution (1 μg/ml, Roche). Images were acquired with an Olympus BX53 microscope equipped with a DP80 camera.

#### Heart Tissue

Hearts were collected after PBS perfusion through the left ventricle, washed in cold PBS and incubated 24 h in 4% paraformaldehyde-PBS followed by incubating for another 24 h in 30% Sucrose-PBS at 4°C. Tissues were embedded in OCT and 15 μm sections were cut and kept in PBS at 4°C until used for immunohistochemical staining. First, cardiac sections were permeabilized with 0.1% TritonX-100 (Sigma-Aldrich) in PBS for 5 min, washed 3 times in PBS and blocked using 5% bovine serum albumin (BSA) together with 2% FBS in PBS for 30 min at room temperature (RT). Successively, golden syrian hamster anti-gp38 primary antibody (clone: 8.1.1., eBioscience; 1:50) was incubated overnight at 4°C. Sections were then washed three times in PBS and incubated with anti-hamster Alexa Fluor 546 secondary antibody (1:500, Thermo Fischer Scientific) for 45 min. Finally, after 3 washings in PBS, DAPI was used to label nuclei. Stained sections were transferred onto microscope slides and mounted with Vectashield mounting medium (Vector Laboratories). Images were acquired with an Olympus BX53 microscope equipped with a DP80 camera.

### Cell Contraction Assay

To assess contractile properties, the Contraction Assay Kit (Cell Biolabs) was used following the manufacturer's protocol. Cells were cultivated with or without 10 ng/mL TGF-β for 72 h and re-seeded in collagen gels for further 72 h. Each condition was analyzed in triplicates or quadruplicates. Images were taken at time 0, 24, 48, and 72 h after re-seeding in a collagen gel. Areas of the gels were measured by ImageJ. Percentage of contraction of all conditions was measured compared to the average of unstimulated cells at day 0.

### Bromodeoxyuridine (BrdU) ELISA Proliferation Assay

Cells were seeded in 96-well plates (5,000 cells/well), stimulated with 10 ng/mL TGF-β for 24 h and BrdU was added to the culture medium for the next 24 h. BrdU incorporation was measured by Colorimetric Cell Proliferation ELISA (Roche) according the manufacturer's protocol. Briefly, cells were fixed with a fixative/denaturing solution for 30 min and further incubated with anti-BrdU-POD antibodies for 90 min at room temperature followed by addition of the substrate solution. Absorbance was measured at 450 nm with correction at 690 nm with the Synergy HT microplate reader (BioTek). Each sample was measured in quadruplicates. Mean absorbance was calculated and unstimulated cells served as control. All experimental conditions were calculated as fold change to controls.

### Caspase Glo 3/7 Assay

For the detection of apoptosis, Caspase 3/7 activity was determined by using the Caspase-Glo® 3/7 Assay Systems (Promega) following the manufacturer's protocol. Briefly, Lin-gp38^+^ cells were seeded in 96-well plates (5,000 cells/well), and stimulated with 10 ng/mL TGF-β for 24 h. Next, cells were incubated with the Caspase-Glo® reagent for 2 h in the dark at room temperature. Luminescent signals were measured with the Synergy HT microplate reader (BioTek). All conditions were analyzed in quadruplicates. Mean luminescence was calculated and unstimulated cells served as control. All experimental conditions were calculated as fold change to control.

#### Statistics

Statistical significance of non-normally distributed data was analyzed by non-parametric Mann-Whitney *U*-test or Kruskal-Wallis test. For Western Blot data analysis, Dunn's *post-hoc* test was used for multiple comparisons. For ELISA data analysis, paired *t*-test was used. For contraction assays, all time-points were compared to each other using Sidak's multiple comparison test. All analyses were computed using the GraphPad Prism 8 software. Differences were considered statistically significant for *p* < 0.05; *n* refers to the number of biological replicates.

## Results

### Identification of a Stromal Cell Subset in the Mouse Heart

**Figure 1:**
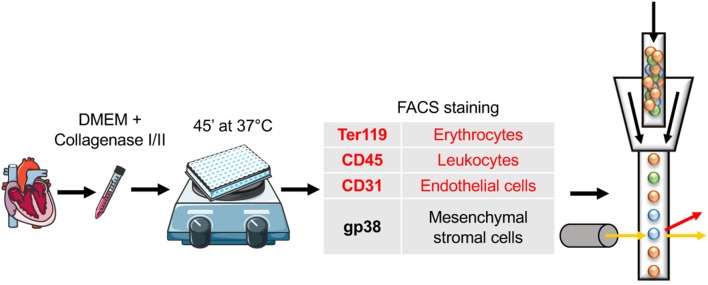
Schematic protocol for stromal cell isolation from the mouse heart. Adult C57BL/6 mice were sacrificed and PBS perfusion via left ventricle was performed. Subsequently, hearts were cut with scissors and digested enzymatically, using pure DMEM complemented with a mixture of collagenase I, II, and DNAse. The incubation was performed for 45′ at 37°C in the presence of magnetic beads on a magnetic stirrer. Next, flow cytometry staining of antibodies included in the panel for stromal cell subset identification was implemented. Markers in red were used for the negative selection (Ter119, CD45, and CD31). In addition single cell suspension was evaluated for the expression of gp38 (in black). Cartoons adapted from SERVIER MEDICAL ART (https://smart.servier.com/), Creative Commons Attribution 3.0 License (https://creativecommons.org/licenses/by/3.0/legalcode).

**Figure 2:**
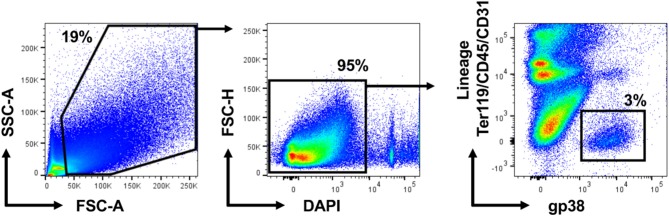
Cardiac Lin^−^gp38^+^ represents a distinct stromal cell population in the mouse heart. Representative pictures and quantification of flow cytometry analysis of hearts from wild-type C57BL/6 mice. Mice of mixed gender were sacrificed, hearts were harvested and processed as described in Figure 1. Cellular debris below 50 k in FSC-A were excluded in SSC-A, FSC-A plots. After that, live cells were selected as DAPI-negative in FSC-H/DAPI plots. The percentage of Lin^−^gp38^+^ cells was quantified from the population of live cells (gated as DAPI-negative in FSC-H/DAPI plots) (*n* = 5).

To isolate fibroblasts from the whole heart of wild-type mice, we established a protocol suitable for obtaining a single cell suspension to process and analyse by two-color flow cytometry. The single cell suspension was first gated in SSC-A, FSC-A plots to exclude cellular debris (below 50 k in FSC-A). Afterwards, live cells were selected as DAPI-negative in FSC-H/DAPI plots. In our two-color strategy, we combined antibodies against the stromal cell marker gp38 conjugated with one fluorescent dye (color 1) with three antibodies against markers of erythrocytes (Ter119), leukocytes (CD45) and endothelial cells (CD31) conjugated with another fluorescent dye (color 2). Anti-Ter119, anti-CD45 and anti-CD31 are thereafter referred to as lineage markers. Two-color flow cytometry analysis of cardiac tissue obtained from a healthy mouse showed a distinct population of gp38^+^ cells negative for lineage markers called further Lin^−^gp38^+^ (Figure 1). Subsequently, we assessed the expression of gp38 in rest of the cell suspension. Lin^−^gp38^+^ cells represented a rare, but distinct population in hearts of wild-type mouse (median 4.2%, Q_1,3_ 3.4%, 4.9% for *n* = 5 of positive cells gated on live, single cell total population; Figure 2).

### Cardiac Lin^−^gp38^+^ Cells Represent Type I Collagen-Producing Fibroblasts

**Figure 3:**
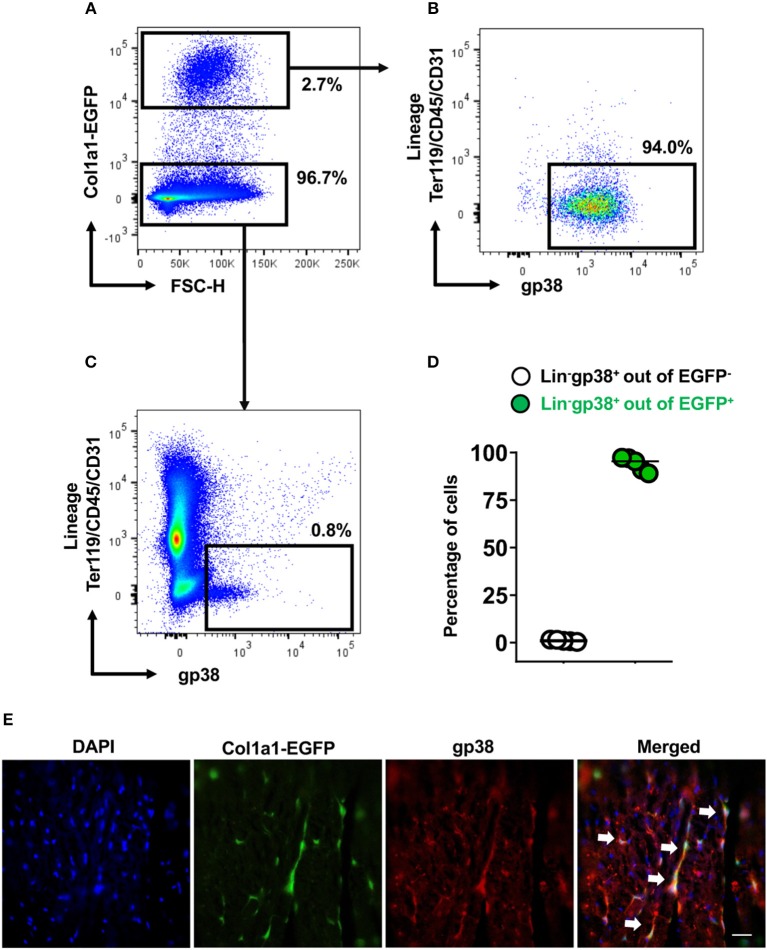
Cardiac Lin^−^gp38^+^ cells represent collagen I producing cells. Representative flow cytometry analysis of cells isolated from hearts of Col1a1-EGFP tg mice. Cellular debris below 50 k in FSC-A were excluded in SSC-A/FSC-A plots. Live cells were first gated as Col1a1-EGFP^+^ or Col1a1-EGFP^−^ **(A)** and then analyzed for expression of Lin and gp38. The percentage of Lin^−^gp38^+^ cells was quantified from Col1a1-EGFP^+^ cells **(B)** and Col1α1-EGFP^−^ cells **(C)**. **(D)** Shows quantification of Lin^−^gp38^+^ cell percentages from EGFP^−^ (white circles) and EGFP^+^ (green circles) cells (*n* = 5). **(E)** Shows representative staining of gp38 (red) and EGFP expression (green) indicating the collagen I-producing cells in the cryosections from hearts of Col1a1-EGFP tg mouse. DAPI stains cell nuclei. Bar = 50 μm.

**Figure 4:**
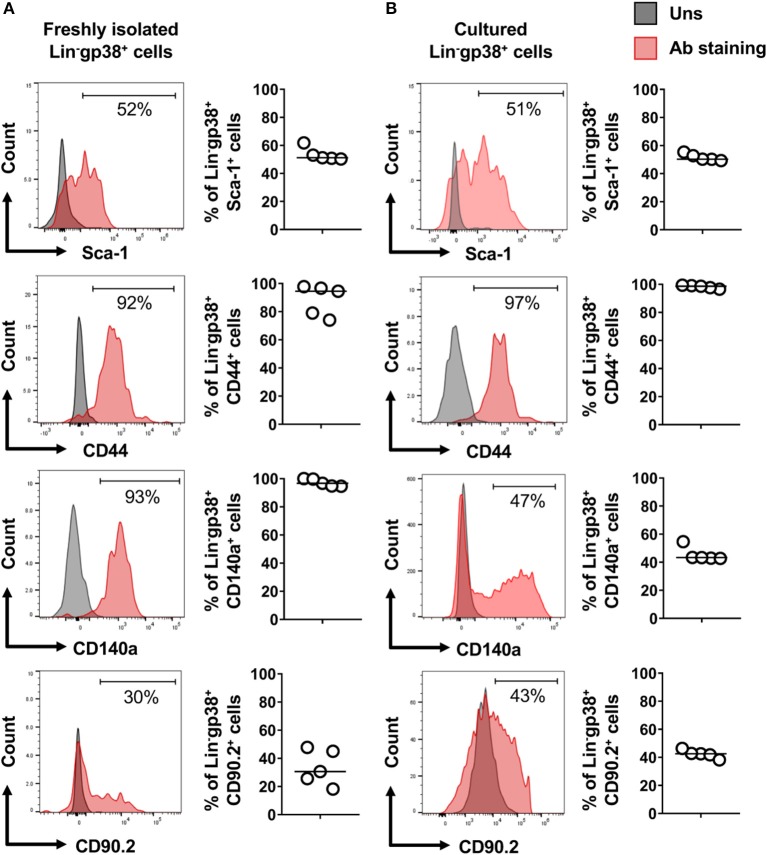
Expression of mesenchymal cell markers on cardiac Lin^−^gp38^+^ cells. Representative flow cytometry histograms (data are representative for 1 out of 5 independent experiments; gray color indicates unstained controls) and quantification of the indicated mesenchymal markers on Lin^−^gp38^+^ cells of single cell suspension obtained from mouse heart **(A)** and cardiac Lin^−^gp38^+^ cells expanded *in vitro* for 3 passages **(B)** (*n* = 5).

Fibroblasts are characterized by collagen I production. In the next step, using Col1a1-EGFP reporter mice, we analyzed whether cardiac Lin^−^gp38^+^ cells produce collagen I. To this aim cardiac cells of Col1a1-EGFP mouse were stained with the two-color panel and analyzed by flow cytometry. As shown in Figure 3, the vast majority of EGFP-positive cells were Lin^−^gp38^+^ (median 95.4%, Q_1,3_ 90%, 97% for *n* = 5, Figures 3B,D). Importantly, Lin^−^gp38^+^ cells were absent in the EGFP-negative population (median 1%, Q_1,3_ 0.6%, 1.6% for *n* = 5, Figures 3C,D). These results demonstrated that type I collagen-producing cardiac fibroblasts could be specifically identified using indicated two-color panel and flow cytometry analysis. Moreover, we performed immunofluorescence staining for gp38 on heart sections from Col1a1-EGFP reporter mice. The pictures presented in Figure 3E show that most of EGFP^+^ cells co-express gp38, and are localized between cardiomyocytes. In the next step, we further characterized cardiac Lin^−^gp38^+^ cells for expression of selected mesenchymal markers using flow cytometry. We found that the majority of Lin^−^gp38^+^ cells were positive for CD44 (median 94.5%, Q_1,3_ 76.5%, 97.2% for *n* = 5) and CD140a (median 96.7%, Q_1,3_ 94.7%, 99.8% for *n* = 5). Furthermore, our data demonstrated that only a subset of cardiac Lin^−^gp38^+^ cells expressed Sca-1 antigen (median 51.2%, Q_1,3_ 50.5%, 57.4% for *n* = 5) and CD90.2 (median 30.6%, Q_1,3_ 21.9%, 46.4% for *n* = 5, Figure 4A). These results confirm previous findings that cardiac fibroblasts are phenotypically heterogeneous.

### TGF-β1 Turns Cardiac Lin^−^gp38^+^ Cells Into Myofibroblast-Like Cells

**Figure 5:**
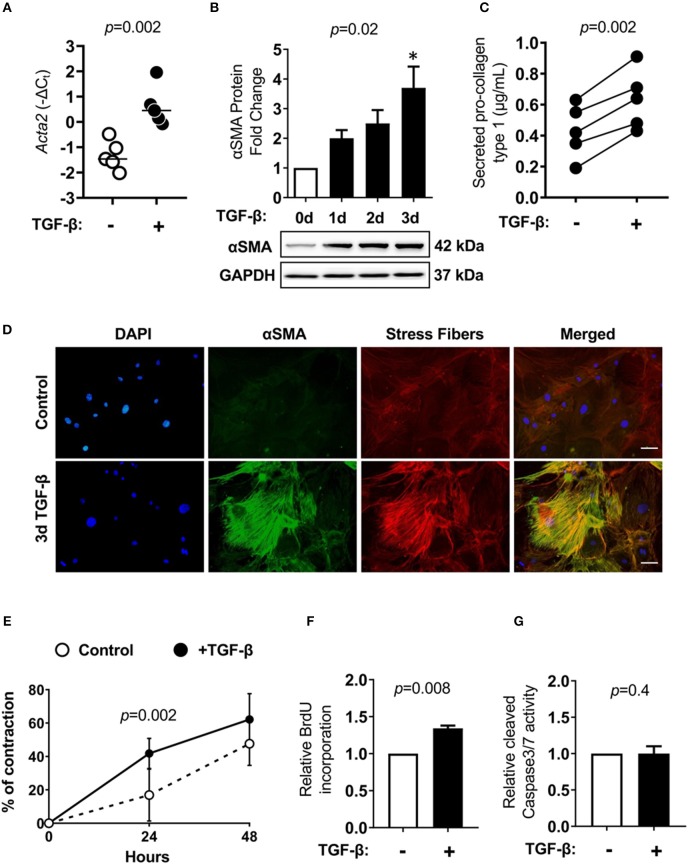
Cardiac Lin^−^gp38^+^ cells response to TGF-β1 stimulation. Cardiac Lin^−^gp38^+^ cells were isolated from hearts of C57BL/6 mice as described in Figure 1 and expanded *in vitro*. **(A)** Shows *Acta2* mRNA expression in cardiac Lin^−^gp38^+^ cells cultured in the presence or absence of TGF-β1 (10 ng/mL) for 24 h (*n* = 5, *p*-value calculated with Mann-Whitney *U*-test). **(B)** Shows quantification and representative immunoblots for αSMA protein levels in lysates of Lin^−^gp38^+^ cells cultured in the presence or absence of TGF-β1 (10 ng/mL) for the indicated time [*n* = 5, *p*-values computed using Kruskal-Wallis test followed by the Dunn's multiple comparisons test, ^*^p < 0.05 (*post-hoc* test vs. control)]. In **(C)**, results from ELISA for pro-collagen type 1 are presented. Lin^−^gp38^+^ cells were seeded at passage 3 and treated with or without TGF-β1 (10 ng/mL) for 7 days. Supernatants were used to assess the amount of pro-collagen type 1 secreted from Lin^−^gp38^+^ cells (*n* = 5, *p*-value calculated with paired *t*-test). **(D)** Shows representative staining of αSMA (green) and phalloidin (red) in Lin^−^gp38^+^ cells cultivated in the presence or absence of TGF-β1 (10 ng/mL) for 3 days. DAPI stains cell nuclei. Bar = 50 μm. **(E)** Shows quantification of Lin^−^gp38^+^ cells contractility precultured in the presence or absence of TGF-β1 (10 ng/mL) for 3 days (*n* = 6, *p*-value calculated with Sidak's multiple comparison test). **(F)** Indicates quantification of BrdU incorporation by Lin^−^gp38^+^ cells cultured in the presence or absence of TGF-β1 (10 ng/mL) for 24 h (*n* = 5, *p*-value calculated with Mann-Whitney *U*-test). **(G)** Shows quantification of cleaved caspase 3/7 activity in Lin^−^gp38^+^ cells cultured in the presence or absence of TGF-β1 (10 ng/mL) for 24 h (*n* = 4, *p*-value calculated with Mann-Whitney *U*-test).

In the next step, we established *in vitro* cultures of cardiac Lin^−^gp38^+^ cells. To this aim, Lin^−^gp38^+^ cells were sorted by FACS from the heart of adult wild-type mice and expanded *in vitro* for up to 4 passages. Cultivated Lin^−^gp38^+^ cells showed similar expression of mesenchymal markers upon 4 passages compared to freshly isolated cells for Sca-1 (median 50.2%, Q1,3 49.5%, 52.2% for *n* = 5), CD44 (median 98.1%, Q1,3 96.8%, 99.2% for *n* = 5), CD140α (median 43.3%, Q1,3 42.8%, 51.8%, for *n* = 5) and CD90.2 (median 42.3%, Q1,3 39.1%, 45.0% for *n* = 5, Figure 4B). The profibrotic cytokine TGF-β1 is known to enhance proliferation of cardiac fibroblasts and converse them into myofibroblast. We investigated how sorted and expanded cardiac Lin^−^gp38^+^ cells responded to stimulation with TGF-β1. As shown in Figure 5A, TGF-β1 up-regulated *Acta2* (gene encoding αSMA) expression 24 h after treatment induction. In line with this finding, αSMA protein was significantly up-regulated 72 h after TGF-β1 treatment (Figure 5B). Stimulation with TGF-β1 induced secretion of pro-collagen I by Lin^−^gp38^+^ cells (Figure 5C). Treatment with TGF-β1 induced also formation of well-structured αSMA-positive fibers, which co-localized with stress fibers (Figure 5D). αSMA protein incorporated into stress fibers has been implicated in increased isometric contraction of myofibroblasts. We measured the contraction strength of Lin^−^gp38^+^ cells using a collagen gel contraction assay. As shown in Figure 5E, treatment with TGF-β1 for 72 h increased contraction of Lin^−^gp38^+^ cells. Finally, we assessed the effect of TGF-β1 on proliferation and apoptosis of Lin^−^gp38^+^ cells. We observed increased proliferation of Lin^−^gp38^+^ cells treated with TGF-β1 in comparison to untreated control (Figure 5F). In contrast, TGF-β1 treatment did not affect apoptosis of Lin^−^gp38^+^ cells (Figure 5G). Altogether, these results indicate that TGF-β1 can effectively differentiate Lin^−^gp38^+^ cells into myofibroblasts-like cells.

## Discussion

Several studies have investigated cellular composition of the adult mammalian heart. It is widely accepted that myocytes account for about 35% of the total number of cells in the heart, while endothelial cells, fibroblasts, vascular smooth muscle and myeloid cells represent remaining fractions. Accordingly, endothelial cells account for up to 60%, myeloid cells for up to 10% and fibroblasts for up to 20% based on immunohistochemistry analyses (16, 17). A recent multiparametric flow cytometry analysis suggested that fibroblasts represent about 15% of non-cardiomyocyte cells (16). This analysis was, however, performed on cells obtained from ventricles only. Recently, these methods were overcome by more modern techniques, such as single cell RNAseq, which allowed a better understanding of the cellular frequencies in the heart (18). Fibroblasts seem to constitute the main stromal cell type in the myocardium (19). Nevertheless, the exact number of stromal cells and fibroblasts in particular in the heart is still debated. In fact, diverse studies have demonstrated that fibroblasts are not only phenotypically but also functionally different, according to their origin, localization in the body and tissues. Furthermore, the lack of reliable, specific and exclusive markers for fibroblasts has made their clear identification difficult.

To date, Fibroblast Specific Protein 1 (FSP1), CD90 (also named Thy-1), CD248 and others have been suggested as fibroblast-specific markers (9). However, subsequent studies demonstrated that endothelial cells and hematopoietic cells might share the expression of these markers. Moreover, several cell types might acquire or loose markers during disease-driven differentiation mechanisms. Kong et al used GFP-driven expression by FSP1 promoter in cardiac homeostasis and disease. The authors demonstrated that following myocardial infarction, half of the FSP1^+^ cells were identified as hematopoietic cells and many endothelial cells were also positive for FSP1, indicating that FSP1 could not be considered exclusive for fibroblasts. CD90 was shown to be expressed in various cell types, including fibroblasts, ovarian cancer cells, endothelial cells, neurons, and hematopoietic cells (20). A recent study of single cell analyses revealed the up-regulation of fibrotic gene profiles: *Postn, Acta2, Adam12, Lox, Wisp1*, and *Ddr2* during cardiac remodeling in response to angiotensin II and after myocardial infarction (21, 22). Lineage tracing studies revealed that periostin-expressing myofibroblasts play a pivotal role during healing and fibrosis in the heart following myocardial infarction. These periostin-expressing myofibroblasts originated from Tcf21-expressing tissue-resident fibroblasts (21). However, the lack of specific monoclonal anti-mouse periostin antibodies, disables the use of this marker for the efficient isolation procedure.

Gp38 (podoplanin) is a mucin-type transmembrane glycoprotein receptor (10). This marker was recently described to stain a subset of stromal cells in SLOs. The gp38^+^ stromal cells play a central role in the formation, reorganization and metabolism of ECM in SLOs as well as in liver, dermis and muscles (5) and are crucial in the functional regulation of T and B cell responses during immunity (23). Importantly, gp38^+^ cells have been also identified in non-lymphoid organs such as pancreas, liver and spleen and were associated with chronic inflammation and fibrosis (24). In fibrosis, gp38^+^ cells function as potent producers of ECM, hyaluronan and connective tissue growth factor, which is required for TGF-β-induced proliferation and differentiation into myofibroblasts (24). In mouse models of biliary and parenchymal liver fibrosis and in steatohepatitis, gp38 expression was linked with CD133 on newly characterized stromal cell population, which considerably expanded in all analyzed models of liver injury and returned to baseline levels during regression of inflammation (13). In the context of fibrotic disease, the gp38 marker has been reported to be expressed in CD34^−^gp38^+^CD90^+^ reticular cells in the skin of the patients with systemic sclerosis and in mouse skin wounds (25) as well as a subset of ADAM12^+^gp38^+^PDGFRα^+^ in skin and muscle acute injury stromal cells (5).

In this study, we demonstrated that lineage (Ter119, CD45 and CD31)-negative and gp38-positive cells represented a population of type I collagen-producing fibroblasts in the adult mouse heart. Accordingly, our results showed that the two-color staining strategy could successfully replace the use of Col1a1-EGFP reporter mice allowing for analysis of cardiac fibroblasts in non-transgenic mice. The two-color staining can be also used to isolate cardiac fibroblasts by FACS. Our proof-of-concept experiment confirmed that these cardiac Lin^−^gp38^+^ cells can be expanded *in vitro* for up to 4 passages and respond to TGF-β1 stimulation by inducing a myofibroblast-like phenotype. Currently, a commonly used method to isolate cardiac fibroblasts is based on differential adhesive properties of isolated cells. In this method, cardiac tissue is disintegrated by enzymatic digestion and obtained single cell suspension is plated onto a cell culture dish. Adherent (typically after 90 min) and proliferating (after days/weeks) cells showing a spindle shape phenotype are considered as fibroblasts (26). In this method no specific cell selection is performed, which may lead to impurity of isolated cells bringing potentially biased outcomes.

Flow cytometry analysis allows for the analysis on multiple markers on a single cell level. Our data showed expression of CD44 and CD140a on cardiac Lin^−^gp38^+^ cells. CD44 is a receptor for hyaluronic acid and other ECM ligands expressed on cardiac fibroblast in homeostasis and increased after heart infarction (27–29), while CD140a was demonstrated to be expressed in a sub-fraction of immature stromal cells with the qualities of mesenchymal stem cells (30). It is well-known that cardiac fibroblasts represent rather a heterogeneous cell population. In line with that, we found only a subset of cardiac Lin^−^gp38^+^ cells expressing Sca-1 and CD90, both well-known markers for mesenchymal stromal cells (31). Sca-1 has been used as a marker for fibroblastic cells, displaying a mesenchymal phenotype (32). Moreover, Sca-1 is expressed on cardiac progenitor cell populations, which attenuate the functional decline and adverse remodeling in post-infarction murine models showing beneficial effects for cardiac repair (33, 34). Interestingly, cardiac Lin^−^gp38^+^ cells resulted to be almost completely negative for the expression of CD29 (fibronectin receptor, not shown), which is considered as another mesenchymal marker (27).

Altogether, here we provide an easy and effective protocol for the identification and isolation of living cardiac fibroblast using two-color flow cytometry. We recommend to use this strategy specially to study the fibroblast populations in healthy or (and) diseased heart. We believe that this protocol will be used in various mouse models of cardiovascular diseases.

## Data Availability

The raw data supporting the conclusions of this manuscript will be made available by the authors, without undue reservation, to any qualified researcher.

## Ethics Statement

Animal experiments were performed in accordance with the Swiss federal law and with the Guide for the Care and Use of Laboratory Animals published by the US National Institutes of Health (NIH Publication, 8th Edition, 2011). The Cantonal Veterinary Office in Zurich had approved animal experiments. The Cantonal Veterinary Office in Zurich had approved all animal experiments (animal license number: 001/2017).

## Author Contributions

GK, PB, MS, and MC: substantial contributions to the conception and design of the work, the acquisition, analysis or interpretation of data for the work. GK, PB, MS, MC, and OD: substantial contributions to drafting the work and revising it critically for important intellectual content, providing an approval for publication of the content, agree to be accountable for all aspects of the work in ensuring that questions related to the accuracy or integrity of any part of the work are appropriately investigated and resolved.

### Conflict of Interest Statement

The authors declare that the research was conducted in the absence of any commercial or financial relationships that could be construed as a potential conflict of interest.
